# Dialect Formation in Ghost Bats: Genetic, Geographic and Morphological Drivers of Social and Echolocation Call Divergence

**DOI:** 10.1002/ece3.72797

**Published:** 2026-01-04

**Authors:** Nicola Hanrahan, Kyle N. Armstrong, Christopher Turbill, Anastasia H. Dalziell, Justin A. Welbergen

**Affiliations:** ^1^ Hawkesbury Institute for the Environment Western Sydney University Richmond New South Wales Australia; ^2^ Research Institute for the Environment and Livelihoods Charles Darwin University Casuarina Northern Territory Australia; ^3^ School of Biological Sciences The University of Adelaide Adelaide South Australia Australia; ^4^ South Australia Museum Adelaide South Australia Australia; ^5^ School of Science Western Sydney University Richmond New South Wales Australia

**Keywords:** acoustic communication, bat song, echolocation call, isolation by distance, social vocalisations

## Abstract

Geographical patterns of vocal dialects in bats are poorly understood, despite growing evidence of complex vocal communication systems. We investigated variation in vocalisations recorded at five ghost bat colonies in the Northern Territory, Australia. We calculated genetic and morphological distances among individuals and correlations with geographic distance. We then determined variation within three social vocalisations (‘chirp‐trill’, ‘squabble’, ‘ultrasonic social’) and the ‘echolocation’ call using seven spectrographic measurements. Finally, we tested whether acoustic distance could be explained by genetic, geographic or morphological distance. Geographic and genetic distance were highly correlated, suggesting the occurrence of isolation by distance. All measures of morphological distance were consistent with Bergmann's Rule, except noseleaf shape, which is likely constrained by its role in echolocation. Geographic variation was evident within each of the three social vocalisations and the echolocation call, with the patterns of geographic variation differing among the four vocalisation types. The degree of difference was surprising, given the ghost bat's long‐range seasonal dispersal. Acoustic distance in chirp‐trill and squabble calls was marginally significantly correlated with genetic (and geographic) distance, but these relationships were not significant after controlling for geography. In contrast, ultrasonic social and echolocation calls varied among colonies but showed no significant associations with other metrics, apart from a weak correlation between ultrasonic social distance and forearm length. This supports the view that these ultrasonic calls are under stabilising selection due to their role in foraging and orientation. This study provides the first evidence of dialect formation in megadermatid bats. It highlights the importance of considering multiple vocalisation types and investigating multiple processes in signal evolution. Overall, we found genetic, geographic and morphological distances accounted for some of the variation in acoustic differences among colonies, but further work is needed to investigate other processes that may also contribute to dialect formation in ghost bats.

## Graphical abstract

Dialect formation in the vocal repertoire of the ghost bat *Macroderma gigas* was investigated by comparing acoustic, geographic, genetic and morphological distances among colonies in the Northern Territory Australia. Dialects were detected in all vocalisation types examined, and while genetic (and geographic distance) explained some of the variation seen, further work is needed to investigate what other processes may be contributing to dialect formation. (Photographed by Bruce Thomson).

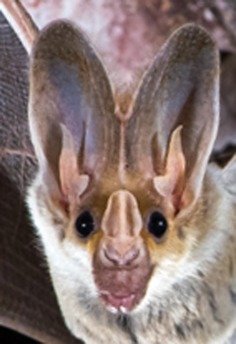

## 1 Introduction

A dialect can be defined as variation within an intraspecific vocalisation that is greater among individuals who are geographically distant than neighbouring individuals (Krebs and Kroodsma 1980). Dialect features are shared within localities and can be characterised by gradual or sharp transitions in vocal parameters among localities (Podos and Warren 2007; Catchpole and Slater 2008). Dialects were originally studied in song birds (oscine passerines) (Marler 1952; Marler and Tamura 1964; Thorpe 1958), and have since been observed in a range of non‐oscine bird species (Kroodsma 2004) as well as non‐avian taxa such as toothed and baleen whales (Deecke et al. 2000; Filatova et al. 2017; Leão et al. 2016; Rendell et al. 2012; Sharpe et al. 2019; Van Cise et al. 2018; Weilgart and Whitehead 1997), pinnipeds (Casey et al. 2018; Le Boeuf and Petrinovich 1974), primates (Cheyne et al. 2007; De La Torre and Snowdon 2009; Hafen et al. 1998; Halloran and Mancz 2015; Maeda and Masataka 1987; Mitani et al. 1992; Wich et al. 2012; Winter 1969; Zürcher and Burkart 2017), fish (Albrecht 1981; Charrier et al. 2013; Danley et al. 2012; Parmentier et al. 2005), rodents (Eiler and Banack 2004; Slobodchikoff and Coast 1980), lagomorphs (Somers 1973), amphibians (Lukanov et al. 2014), hyraxes (Kershenbaum et al. 2012) and bats (Esser and Schubert 1998; Jiang et al. 2010; Knörnschild, Nagy, et al. 2012; Prat et al. 2017).

Several theories have been proposed to explain geographical variation of vocalisations within species (Podos and Warren 2007). Environmental factors such as vegetation structure, noise or climatic variables can result in vocalisation structures that optimise transmission in local conditions (Boncoraglio and Saino 2006; Potvin and Clegg 2015). In vocalisations involved in mate‐choice, sexual selection can also result in divergence among and convergence within populations. For example, female yellowhammers ( *Emberiza citrinella* ) exhibit assortative mating with males of their own local dialect (Baker et al. 1987). Drift can also lead to divergent vocalisation structures among geographically distant populations. Dialects can form when genetic drift affects genes controlling morphological structures involved in sound production or in the parts of the brain that are important for signal formation (Armstrong and Coles 2007), or when cultural drift results in the accumulation of mistakes introduced in vocalisations acquired via social learning (Janik and Slater 1997; Nottebohm 1972, 1991). In most cases, a combination of factors likely affects the formation of dialects and often, differences can be reinforced by assortative mating (Jiang, Bolnick, and Kirkpatrick 2013; Jiang, You, et al. 2013).

Bats are an important model of vocal communication because they have complex vocal repertoires that include songs that attract mates and defend territories (Bohn et al. 2013; Hanrahan 2019; Knörnschild et al. 2017; Smarsh 2015). Bats provide a particularly interesting model of dialect formation because of the dual use of sound production in this group, for both spatial orientation and communication (Fenton 2003). All echolocating bats rely on high‐frequency sound for spatial awareness, prey detection and localisation in complete darkness (Jones and Teeling 2006). In recent years, the use of echolocation calls for communication has also been described in several species (Jones and Siemers 2011; Knörnschild, Jung, et al. 2012). Research on dialects in bats has concentrated mostly on echolocation calls. Geographic variation in echolocation call structures has been attributed to differences in prey species, acoustic interference from sympatric species, climatic conditions or habitat structure (Jacobs et al. 2017; Jiang, Bolnick, and Kirkpatrick 2013; Jiang, You, et al. 2013; Law et al. 2002). Others have suggested that cultural drift is the process driving geographic variation because echolocation calls also have a function in communication and develop at least in part via social learning (Sun et al. 2013; Jiang et al. 2010; Jacobs et al. 2017). For example, Hiryu et al. (2006) showed convergence of echolocation structure to that of conspecifics when a new individual was added to a colony. In other species, geographic variation in echolocation calls appears to be an outcome of genetic drift (Armstrong and Coles 2007; Armstrong and Kerry 2011; Yoshino et al. 2008), while some studies have failed to find any clear determinants of geographic variation and suggested a combination of ecological and social factors as the causes of observed patterns (Lin et al. 2015).

In contrast to work on echolocation calls, studies investigating geographic variation in bat social vocalisations are few, despite the potential to greatly increase our understanding of how cultural and genetic evolution drive dialect formation in bats. There is increasing evidence that bats can develop social vocalisations through vocal production learning (Boughman 1998; Esser 1994; Janik and Knornschild 2021; Prat et al. 2017), and so in theory bat social vocalisations could vary geographically to a similar extent to what has been reported in birds (e.g., Podos and Warren 2007). However, the few studies that have investigated geographic variation in bat social vocalisations (Boughman 1998; Davidson and Wilkinson 2002; Esser and Schubert 1998; Lin et al. 2022; Prat et al. 2015, 2017) with a handful of notable exceptions (Montero et al. 2018; Sun et al. 2020; Zhang et al. 2024) do not control for genetic variation, making it difficult to determine if observed patterns of variation are a result of genetic or cultural evolution.

The ghost bat *Macroderma gigas* (Megadermatidae) is a highly vocal species that produces a wide array of human‐audible and ultrasonic social vocalisations, in addition to a high‐frequency echolocation call (Guppy et al. 1985; Hanrahan 2019; Hanrahan et al. 2019, 2022, 2024). The ghost bat is endemic to Australia and forms colonies in caves and old mine workings (Churchill 2008). Studies of the population structure of ghost bats across their northern Australian range (Worthington Wilmer et al. 1994, 1999) found an absence of contemporary gene flow among geographically isolated populations (‘isolation by distance’: Wright 1943), which contrasts with evidence for high gene flow in other bat species (Burland et al. 1999; Kerth et al. 2003) but may be attributed to the rarity of suitable roost sites and high fidelity to natal sites by females (Worthington Wilmer et al. 1994, 1999). Ghost bats in the Northern Territory (NT) showed intermixing between colonies that are located < 40 km apart (Pine Creek and Claravale Station), but exhibited no evidence of mixing between these colonies and a Kakadu National Park‐based colony located c. 200 km away (Worthington Wilmer et al. 1999). Thus, it is possible that this limited long‐distance movement has led to divergence of some social vocalisation characteristics among isolated groups and resulted in the formation of dialects. While there is no evidence yet of vocal learning in this species, nor in the Megadermatidae family more broadly, the ghost bat's colonial roosting behaviour provides opportunities for individuals to learn from each other. Further, the complexity of the ghost bat's vocal repertoire (Hanrahan 2019), including a song‐like complex vocalisation predominantly produced by males (Hanrahan et al. 2022), means that any study of social vocalisations in ghost bats should take into account the possibility that some ghost bat vocalisations are learnt socially. In sum, the tendency for limited intermixing among distant colonies and the use of complex acoustic communication make the ghost bat an interesting model for investigating patterns and drivers of geographical variation in vocalisations.

In this study, we assessed the extent of genetic, morphological, and acoustic divergence among five geographically dispersed ghost bat colonies within the NT. Genetic divergence was calculated using single nucleotide polymorphisms (SNPs) and four morphometrics (forearm, ear, tragus and nose leaf) were examined. We investigated geographic variation within the ‘chirp‐trill’, ‘squabble’ and ‘ultrasonic social’ vocalisations used in social interactions, and the ‘echolocation’ call, which is used for orientation (Hanrahan 2019; Hanrahan et al. 2022). The acoustic structure of echolocation calls in bats is optimised to the level of clutter in which a species hunts and the prey that it targets (Aldridge and Rautenbach 1987; Fenton 1990), and so they are under strong, stabilising selection mediated by the environment (Jones and Holderied 2007). As there is no evidence that ghost bats in different regions have different prey preferences or forage in different environments, we predicted very little geographic variation in the ghost bat's echolocation call. The ultrasonic social vocalisation is produced during social interactions, but like the echolocation call, it is produced nasally. Peaks in the production of this call during the parturition period suggest it may be involved in mother‐pup communication (Hanrahan et al. 2019). The ultrasonic social vocalisation has structural similarities with the ghost bat's echolocation call (Hanrahan 2019), and therefore the structure of the ultrasonic social vocalisation may be constrained by stabilising selection on the echolocation call—even if the two vocalisations have separate functions—because they share the same vocal production mechanisms. The chirp‐trill and squabble are social vocalisations in the human audible range that are hypothesised to function as a contact and an agonistic vocalisation, respectively (Guppy et al. 1985; Hanrahan et al. 2022; Tidemann et al. 1985). Both vocalisations are emitted orally, and thus, it is unlikely that selection on the echolocation call would constrain the structure of these calls. We predicted that due to geographic distance between colonies, (genetic) isolation by distance will have occurred, subjecting the chirp‐trill and squabble to drift. To test these predictions, we first examined the degree of connectivity among five distant NT colonies of varying geographic isolation by quantifying genetic and morphological divergence. Second, we determined the degree of acoustic divergence of the four vocalisation types among the five colonies. Third, we assessed whether genetic and morphological divergence was correlated with acoustic distance among colonies by modelling each of the four vocalisation types separately. Our results provide insight into the function of the four vocalisation types and the drivers of dialect formation in ghost bats.

## 2 Materials and Methods

### 2.1 Study Species and Location

**FIGURE 1:**
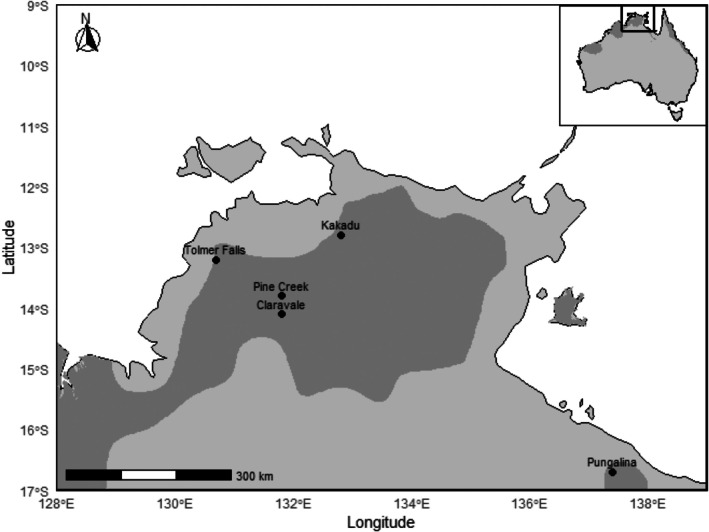
The main map shows the location of the five study sites in the Northern Territory, Australia. The dark grey polygons are the current estimated range of the ghost bat (Australasian Bat Society—BatMap 2024). The inset map shows the location of the study area in relation to the rest of northern Australia.

Five ghost bat maternity colonies located in the NT, Australia were used for this study (Figure 1; Table 1): Pine Creek, Kakadu National Park (‘Kakadu’), Tolmer Falls, Claravale Station (‘Claravale’) and Pungalina‐Seven Emu Sanctuary (‘Pungalina’). The first four sites form a geographic cluster in the northernmost section of the NT (Figure 1). Pungalina is an outlier geographically, located between 654 and 810 km southeast of the other sites (see Table S1 for distances between each site). This study focuses on colonies in maternity roosts (as opposed to sporadically used day‐roosts) because large numbers of individuals gather at maternity roosts during the breeding season when resources are abundant (Toop 1985). Therefore, maternity roosts are likely to best represent the acoustic variability present in each area of focus.

**TABLE 1:** Location of study sites and the number of adult ghost bats from which genetic and morphometric data were collected. Tissue and measurements were collected from males and females.

Site name	Genetic data	Morphometric data	Lat (° S)	Lon (° E)
	No. of individuals			
Pine Creek	26^a^	36	13.8	131.8
Claravale	7	22	14.1	131.8
Tolmer Falls	33	55	13.2	130.7
Kakadu	9	16	12.8	132.8
Pungalina	4^b^	12	16.7	137.4

^a^ Includes 23 individuals trapped and 3 carcasses found in roost.

^b^ Includes 3 individuals trapped and 1 carcass found in roost.

### 2.2 Tissue Collection, Phylogenetic Sequencing and Genetic Distance

Seventy‐five ghost bats were captured approximately 100 m from their known maternity roosts in December 2016 and July 2017 using a 12 m nylon mist net. Bats were lured into the net by broadcasting a playback track containing full‐spectrum recordings of squabble and ultrasonic social vocalisation exemplars from all of the five colonies for this study (for full details see: Hanrahan et al. 2024). Once trapped, each bat was removed from the net immediately and placed into a calico bag until the completion of the catching period and associated experiment (see Hanrahan et al. 2024). During processing, each ghost bat was held face up with its wing extended over a sterilised board by an experienced bat handler. A second trained person then collected two 3 mm tissue biopsies from each captured bat—one from the wing and one from the tail membrane—taking care to avoid veins and connective tissue. Tissue biopsies are an effective method for obtaining genetic samples from bats and the resulting hole has been shown to completely heal within 4 weeks (Worthington Wilmer and Barratt 1996). Membrane biopsies from each individual were placed into a 2 mL sample vial containing either 100% ethanol or indicating silica beads. These tissue samples from live bats were supplemented with samples taken from bat carcasses opportunistically found within roosts during routine acoustic equipment maintenance (3 individuals from Pine Creek and 1 individual from Pungalina), bringing the total number of individuals in the genetic dataset to 79. Samples were refrigerated in the field using a Waeco car fridge (CFX‐40 W) before being transported back to Western Sydney University on ice where tissue stored in ethanol was kept frozen at—18°C whereas tissue stored in silica was refrigerated at 4°C. All tissue biopsies were suspended in 100% ethanol and sent to Diversity Arrays Technology for DNA extraction and sequencing.

Single nucleotide polymorphism (SNP) datasets were constructed using the DaRTseq method. DaRTseq (RADseq) is a genome complexity reduction technique that involves the intelligent selection of the genome fraction corresponding predominantly to active genes (Jaccoud et al. 2001). The SNP dataset was a matrix of bi‐allelic genotypes (homozygotes of either one reference allele, or one alternative allele, plus heterozygotes) from a single locus position on each DNA fragment, as provided by Diversity Arrays. Data preparation and analysis were conducted using the dartR package (Gruber et al. 2018), except where specified. The dataset was first checked and filtered to remove loci with a minimum allele frequency of 0.85. One sample sequenced from the desiccated remains of a ghost bat found at Pungalina was removed from the dataset due to poor sample quality. The DArTseq SNP dataset produced 21,467 filtered SNPs from 78 individuals across the study colonies.

Principal Coordinate Analysis (PCoA) ordination was used to visualise the relationship between genotypes and colonies, and then the number of ancestral populations was estimated using individual ancestry coefficients calculated using non‐negative matrix factorisation (sNMF), with values of K (number of populations) varying between 2 and 5 (Frichot and François 2015). Genetic distance was calculated by constructing a matrix of the Fixation index (*F*_ST_), that is, the variance of allele frequencies among the colonies, where values range from 0 to 1 and a higher *F*_ST_ indicates greater genetic diversity between colony pairs (Wright 1969). *F*_ST_ values were calculated in R (3.5.1; R Core Team 2018) using the package StAMPP and the function stamppFst (Pembleton et al. 2013). To determine if colonies that are geographically further apart are also genetically less alike, we conducted isolation by distance analysis by performing a Mantel test (with 9999 permutations) among sites based on geographic distance (calculated from exact coordinates of sites) and genetic distance. Mantel tests were completed using the ade4 package and the mantel.rtest function (Dray and Dufour 2007).

### 2.3 Morphometric Measurements and Morphologic Distance

**FIGURE 2:**
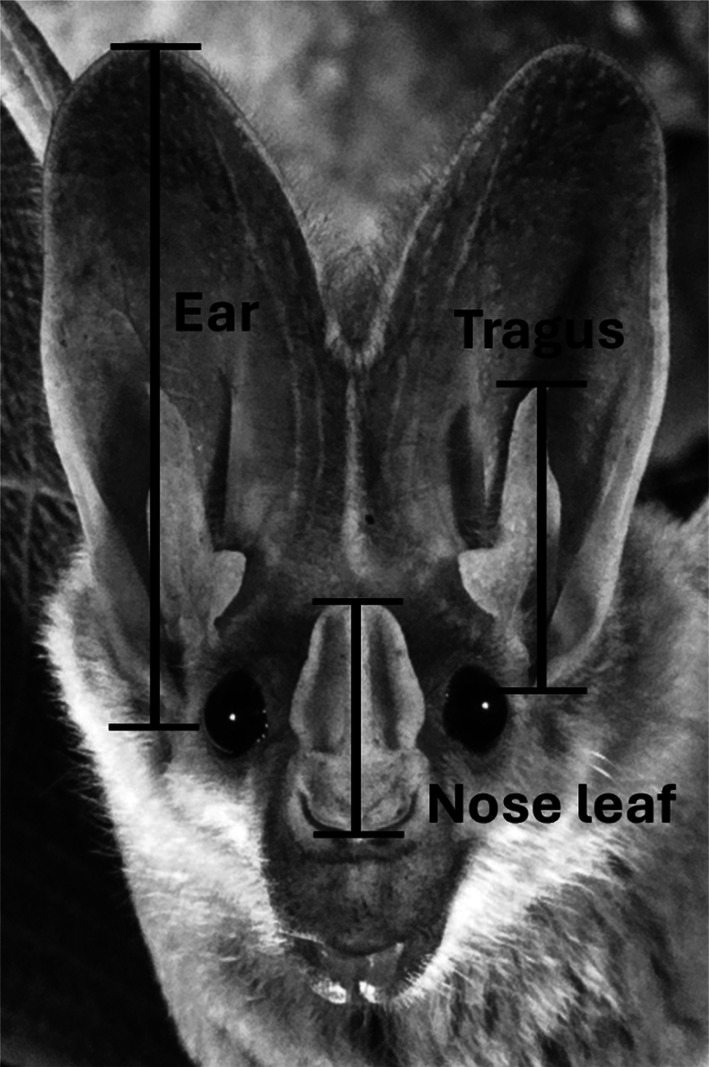
Illustration of the three facial measurements: ear length, tragus height and nose leaf height, collected from captured ghost bats at all five colonies (Photographer: Bruce Thomson).

Four measurements were taken from 141 ghost bats trapped at the five study colonies in December 2016, July 2017 and May 2018 using vernier callipers: forearm length, ear length, tragus height and nose leaf height (Figure 2). Forearm length was preferred as an indicator of body size over body mass because mass can vary seasonally in bats (e.g., Welbergen 2010, 2011). We also measured external morphological features that are involved in the transmission and reception of acoustic signals (ear length, tragus height and nose leaf height). All features were measured from the base of the feature to the highest point (Figure 2).

To evaluate potential sexual dimorphism, measurements of forearm length, noseleaf, ear and tragus were compared between males and females. Individuals with missing data were excluded prior to analysis. For each trait, the mean ± SD was calculated by sex, and Welch's two‐sample t‐tests were used to test for differences in mean values between sexes, which do not assume equal variances. Effect sizes were calculated using Cohen's d to quantify the magnitude of any differences.

The mean value of each morphological feature for each study site was calculated, and the Euclidean distance between each pair of sites for each feature was calculated using the ‘dist’ function in R. The four morphometrics were then condensed into two dimensions using discriminant function analysis (DFA), and Euclidean distance was calculated between the first linear discriminant (LD1) from each study site to assess the overall effect of morphological divergence.

### 2.4 Acoustic Recording, Analysis and Distance Calculation

Ghost bat vocalisations were recorded simultaneously at each of the 5 study sites for a minimum period of 3 months (December 2016—February 2017) and up to 2 years, using Song Meter SM3Bat acoustic recorders fitted with a SMM‐U1 ultrasonic microphone (Wildlife Acoustics, Massachusetts, USA). Calls were recorded in full‐spectrum WAV format at a sampling rate of 256 kHz. Recorders were set to record 24 h per day for two of the sites where the recorder was placed close to the roosting bats inside underground structures (Pine Creek: 100 m inside a disused mine adit; Pungalina: 10 m inside a limestone cave entrance with cluttered vegetation). At the other three sites, access into the cave was restricted, so the recorder was placed at the roost entrance (Claravale: vegetation cluttered, Kakadu: vegetation open) or at a known feeding perch close by (Tolmer Falls: in a gorge with no vegetation). For these latter 3 locations, the recorder was set to record from sunrise to sunset. Each of the 5 units recorded for 5 min every 30 min if vocalisations exceeded the decibel and call length thresholds. Despite variability between recording locations, efforts were made to ensure the recording conditions remained consistent through careful equipment placement.

**FIGURE 3:**
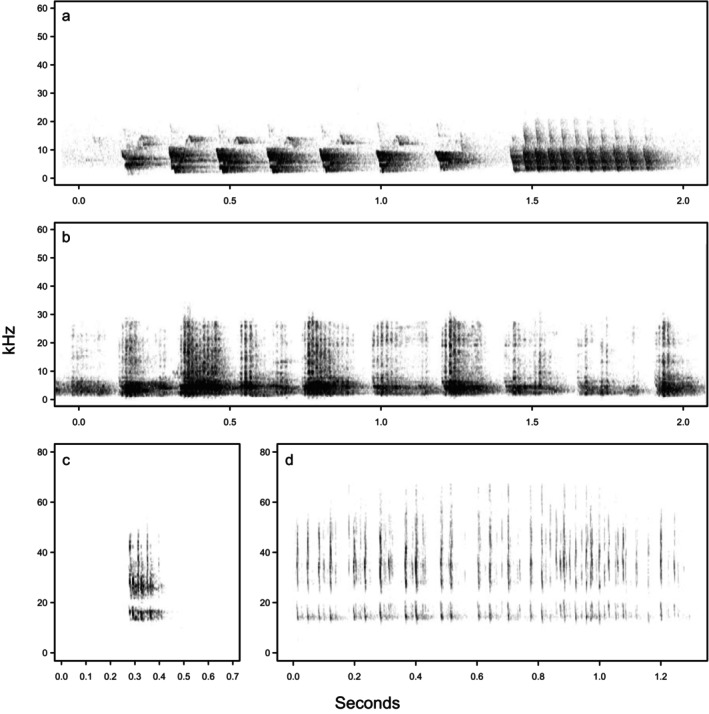
Spectrogram images of the (a) chirp‐trill, (b) squabble and (c) ultrasonic social vocalisations and the (d) echolocation call of the ghost bat.

The vocal repertoire of the ghost bat was defined using an extensive acoustic dataset from the Pine Creek colony that was observed to contain at least 12 distinct social vocalisation types (Hanrahan 2019; Hanrahan et al. 2022). Three social vocalisation types were chosen for the present study: ‘squabble’, ‘chirp‐trill’ and ‘ultrasonic social’, since these are the most commonly recorded vocalisations produced by ghost bats (Hanrahan et al. 2019, 2022, 2024; Figure 3). The ‘echolocation’ call of the ghost bat was also included in this study to allow a comparison between a call type that is generally subject to strong stabilising selection due to its critical role in orientation and prey detection (e.g., Jones and Teeling 2006), and social vocalisations that are presumably less constrained by environmental selection pressures or morphometric features.

A band‐limited energy detector (BLED) was designed in Raven Pro 1.5 (Bioacoustics Research Program, 2014) to automatically select any sounds (detection) within the recordings that met the detector settings. The detector was designed to detect all multi‐element vocalisations from the entire repertoire of ghost bat vocalisation types. Specifically, the detector selected sounds between 2 and 80 kHz, with a minimum duration of 0.02 s, a maximum duration of 5 s and a minimum separation of 0.1 s between elements within a single multi‐element call (i.e., a single detection). Signal‐to‐noise settings were a minimum occupancy of 40% and a signal‐noise ratio of 10 dB. Noise power estimation parameters were a block size of 2 s, hop size of 0.5 s and percentile of 20. Seven spectrographic measurements were collected for each call (Table 2; Figure S1) to allow differentiation among vocalisation types. Spectrographic measurements for each call were calculated in a Hanning sound window of size of 1024 samples and a 50% time grid overlap. Detections were classified into vocalisation types using a purpose‐built discriminant function analysis (DFA) based classifier in R, using a predefined reference set, detections were assigned to a vocalisation type based on the seven variables in table 2 (detailed further in Hanrahan 2019). From the dataset of classified vocalisations, up to 50 exemplars of each vocalisation type (chirp‐trill, squabble, ultrasonic social and echolocation) were chosen from each colony. It was difficult to obtain recordings with high signal‐to‐noise ratios from the Tolmer Falls site (because of the noise from a waterfall nearby), and both the squabble and ultrasonic social were recorded less often at this site and so the sample size for this site was smaller for these vocalisations compared to the chirp‐trill and echolocation.

**TABLE 2:** Acoustic measurement variables calculated in Raven Pro (Charif et al. 2010) that were used to differentiate between ghost bat vocalisation exemplars from different colonies.

Measurement	Description
Bandwidth 90% (BW_90_)	The difference between the frequencies at 5% and 95% of the frequency range of the signal (kHz)
Interquartile range (IQR) bandwidth (BW_IQR_)	The difference between the frequencies at 25% and 75% of the frequency range of the signal (kHz)
Peak frequency (*F*_peak_)	The frequency at which the call was the loudest (kHz)
PFC max frequency (maximum frequency: *F*_max_)	The peak frequency contour traces the path of the loudest frequency from the start to the end of the vocalisation. This variable takes the highest frequency from across the contour as a proxy for maximum frequency in the vocalisation. Maximum frequency cannot be directly calculated when using the batch function in Raven Pro as maximum frequency is specified as the maximum frequency of the selection box (kHz)
PFC min frequency (minimum frequency: *F*_min_)	As above, but minimum frequency is calculated from the peak frequency contour (kHz)
Length	The number of frames contained in a selection. The number of frames equals the number of windows in the selection in one channel (frames)
Peak time	The first time in the selection at which a sample with amplitude equal to Peak Amplitude occurs (s)

For each vocalisation type, each of the seven measured acoustic variables was assessed using a one–way ANOVA (function aov from the stats package: R Core Team 2018) to determine if there were significant differences in that acoustic variable among the study colonies. If the model was significant, a post hoc test (TukeyHSD function from the stats package: R Core Team 2018) was conducted using *p*‐values adjusted for multiple comparisons to identify the colonies that differed from each other based on that specific variable. Discriminant function analysis (DFA; lda function in the MASS package: Venebles and Ripley 2002) was conducted using only the acoustic variables that were found to be informative (significant) in separating the vocalisations among sites (Irwin et al. 2008). One‐way ANOVA and Tukey's pairwise tests were also conducted using the first linear discriminant (LD1) from the DFA to the difference in overall acoustic structure among colonies.

Treating vocalisation types separately, we calculated acoustic distances among colonies using the first two linear discriminants to construct a pairwise squared Mahalanobis distance matrix, representing the distance from each vocalisation exemplar to the centroid of each colony (Yoktan et al. 2011). Mahalanobis distance was chosen over Euclidean distance for multivariate data because it is suitable for non‐spherical symmetric data (McLachlan 2004). To determine if vocalisations could be assigned to their colony of origin using acoustic structure, 50% of the dataset was used as a test dataset, and DFA was used to assign each individual vocalisation to a colony. The percentage of correctly identified selections for each vocalisation type was calculated.

### 2.5 Correlation of Distance Matrices

The degree of correlation between the acoustic distance of each vocalisation and the geographic, genetic and morphological distance datasets was determined using Mantel tests with 9999 permutations. Mantel tests were also run on individual morphometric features to determine if any measurement (forearm length, ear length, tragus height and nose leaf height) was by itself correlated with acoustic, geographic or genetic distance among colonies. Because genetic and geographic distances were themselves highly correlated (indicating isolation by distance), partial Mantel tests were subsequently performed to assess whether correlations between acoustic and genetic distance remained significant after accounting for geographic distance, and vice versa. All tests were conducted using the ade4 and vegan packages in R (Dray and Dufour 2007; Oksanen et al. 2024).

## 3 Results

### 3.1 Genetic Distance

**FIGURE 4:**
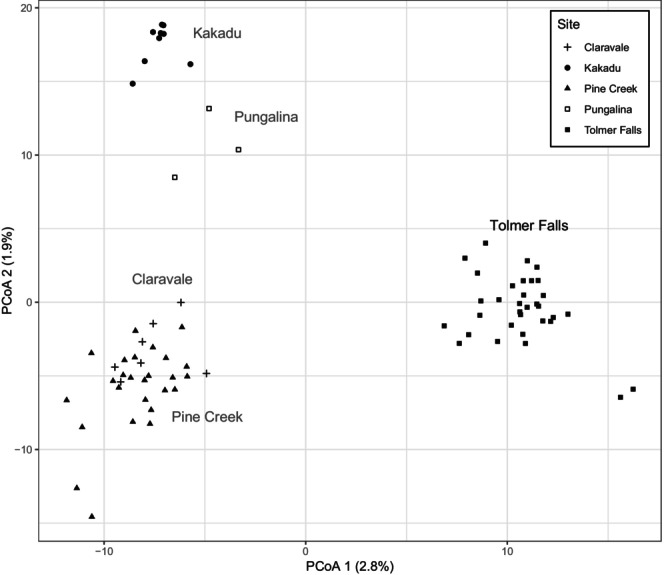
Bivariate scatterplot of PCoA axis 1 and PCoA axis 2 calculated using PCoA ordination on 21,467 filtered SNP loci sequenced from wing biopsies from 78 individuals using DArTseq.

Each of the five ghost bat colonies in the Northern Territory (NT) were clustered separately by the PCoA ordination of the SNP loci, except for Pine Creek and Claravale (Figure 4; 21,467 filtered SNPs from 78 individuals). Ancestral coefficients were estimated for *K* = 2–5 populations (Figure S2). When *K* = 2, clear genetic divergence was evident between the Tolmer Falls colony and the other colonies. When *K* was increased to 3, Claravale and Pine Creek formed one group, Kakadu and Pungalina formed another group, and Tolmer Falls was separate. This grouping was maintained when *K* was increased to 4. When K was increased to 5, individuals from Pine Creek and Claravale continued to cluster together, while Kakadu and Pungalina became separate, and two individuals split away from the remainder in the Tolmer Falls group.

**FIGURE 5:**
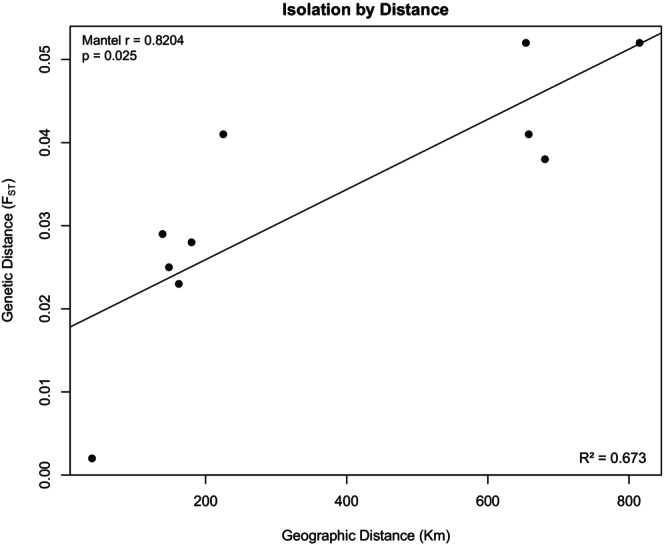
Scatterplot showing the high level of correlation between genetic distance and geographic distance among colonies. The trend line shown here was calculated using linear regression, but correlation analysis was conducted using a Mantel test.

Isolation by distance analysis showed that ghost bat genetic distance was strongly correlated with geographic distance (Mantel test, *r* = 0.8204, *p* = 0.025) (Figure 5). The Pine Creek and Claravale colonies are located 39 km from each other and were genetically indistinct (*F*_ST_ = 0.0020), whereas the Pungalina colony is located 654–815 km from the other colonies and had a relatively high genetic distance from the other colonies (*F*_ST_ = 0.0378 to 0.0523). Despite this, the *F*_ST_ values overall were close to zero suggesting some gene flow among all colonies (Table S1).

### 3.2 Morphological Distance

We assessed sexual dimorphism in external morphology using Welch's two‐sample t‐tests comparing males (*n* = 53) and females (*n* = 86) for forearm length, noseleaf, ear and tragus measurements (Table S2). Females had slightly longer forearms on average (*t* = 3.01, *p* = 0.003; Cohen's *d* = 0.52), but this difference was small in absolute terms (≈1.7 mm, < 2% of mean forearm length). No significant differences were detected between sexes for noseleaf, ear, or tragus dimensions (all *p* > 0.1; |*d*| < 0.3). These results indicate limited sexual dimorphism in external morphology. Given the minor magnitude of the forearm difference, the lack of dimorphism in cranial features more directly related to sound production, and the fact that acoustic recordings were obtained at mixed‐sex roosts rather than from identified individuals, we pooled males and females in subsequent analyses.

**FIGURE 6:**
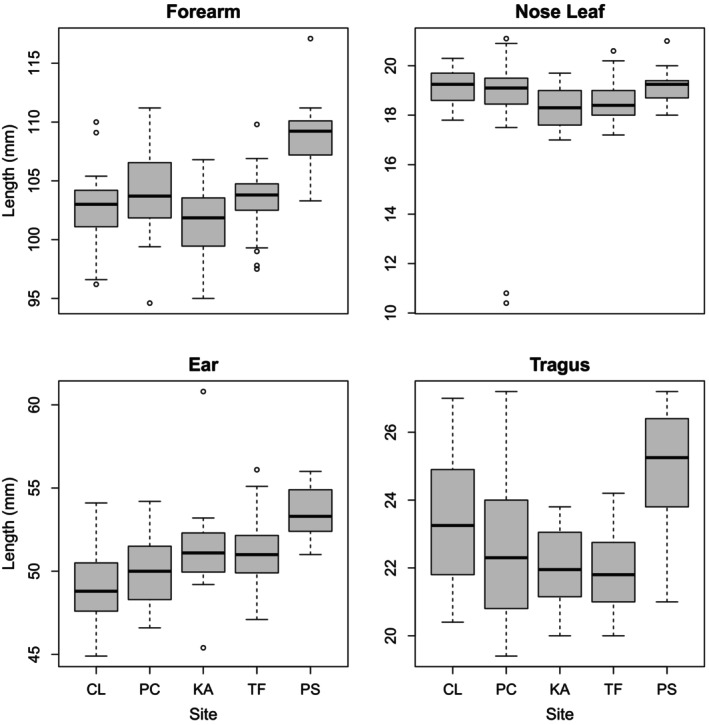
Boxplots illustrating the difference observed in the mean length of forearm, ear and tragus among the four northern colonies (CL = Claravale, PC = Pine Creek, KA = Kakadu and TF = Tolmer Falls) and the Pungalina colony (PS). The sites are listed in geographical order from closest to the Claravale colony to most distant, showing a geographic pattern, particularly in forearm, ear and tragus lengths.

**FIGURE 7:**
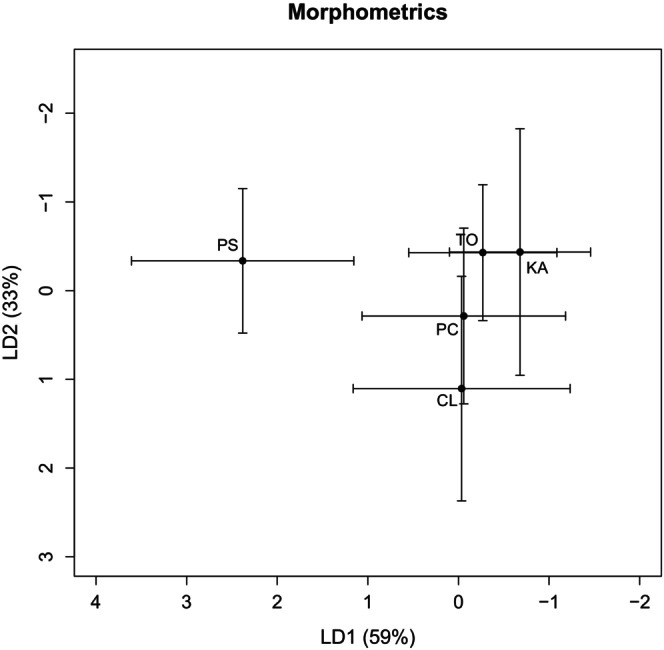
Plot of mean linear discriminant 1 and linear discriminant 2 calculated based on the four morphological indices. The whiskers illustrate one standard deviation from the mean for linear discriminant 1 and linear discriminant 2. Letters indicate ghost bat colonies (CL = Claravale, PC = Pine Creek, KA = Kakadu, TF = Tolmer Falls, PS = Pungalina).

Ghost bats captured at Pungalina had significantly longer forearms, larger ears and larger tragi than the other four colonies (Figure 6, Table S3). Ghost bats captured at Claravale had smaller ears than those at Tolmer Falls and Kakadu, but a larger tragus than those from Tolmer Falls. The length of the nose leaf did not differ significantly among colonies. The DFA performed on all four morphometrics showed that the Pungalina colony is morphologically distinct from the other colonies, while the other colonies are all morphologically similar (*F*_4,136_ = 19.89, *p* < 0.001; Figure 7). LD1 explained 60% of morphological variation and represents predominantly forearm and tragus length. LD2 explained 33% of the variation and represents a mixture of nose leaf, ear and tragus height.

Forearm and ear length were marginally significantly (*p* < 0.1) correlated with both genetic (forearm: *r* = 0.8123, *p* = 0.0937; ear: *r* = 0.7914, *p* = 0.0689) and geographic (forearm: *r* = 0.9800, *p* = 0.0753; ear: *r* = 0.8680, *p* = 0.0578) distance.

### 3.3 Acoustic Distance

Geographic variation was found in all four vocalisation types examined: chirp‐trill, squabble and ultrasonic social vocalisations and the echolocation call.

#### 3.3.1 Chirp‐Trill

**FIGURE 8:**
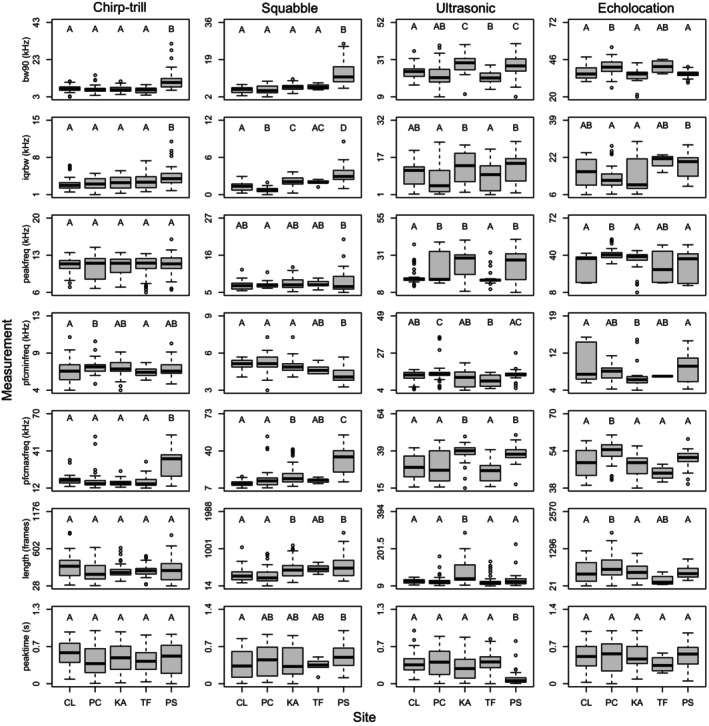
Boxplots of the seven acoustic measurements, Bandwidth 90% (BW_90_), Interquartile range (IQR) bandwidth (BW_IQR_), Peak frequency (*F*_peak_), PFC max frequency (maximum frequency: *F*_max_), PFC min frequency (minimum frequency: *F*_min_), Length, ‘Peak time’ (for details on measurements, see Table 2) used in this study separated by vocalisation type and study site. Each box encompasses the interquartile range. The bars (whiskers) show the first and fourth quartiles. The thick horizontal line is the median value per site while the open circles show outliers in the data. Letters indicate ghost bat colonies (CL = Claravale, PC = Pine Creek, KA = Kakadu, TF = Tolmer Falls, PS = Pungalina).

**FIGURE 9:**
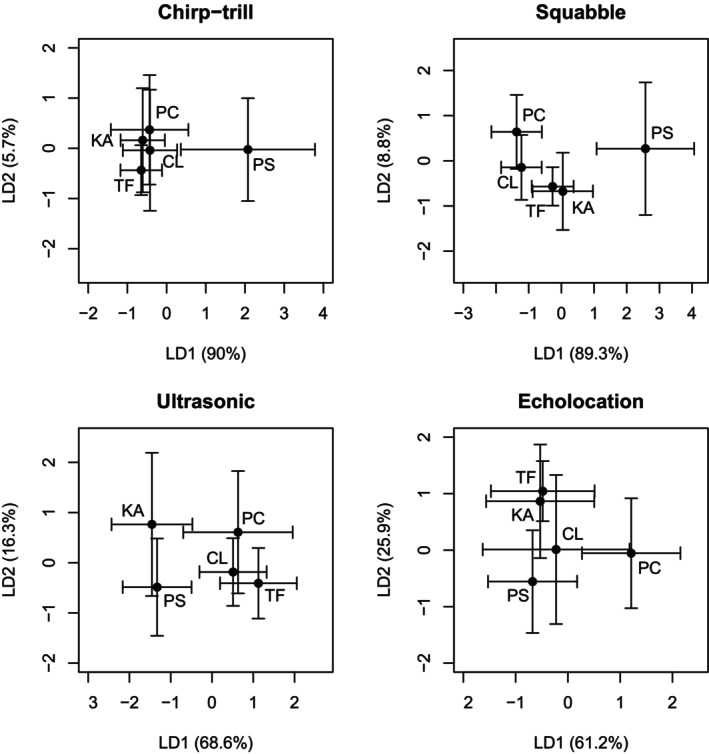
Relationships between acoustic structure at each of the five study colonies based on discriminant function analysis: Pine Creek (PC), Claravale (CL), Kakadu (KA), Tolmer Falls (TF) and Pungalina (PS). Each point shows the mean for LD1 and LD2 for each site. The bars represent one standard deviation from the mean.

Significant geographic variation of the chirp‐trill was found in four of the seven acoustic measurements: BW_90_, BW_IQR_, *F*_min_ and *F*_max_ (Figure 8, Table S4). A DFA of the informative measurements placed Pine Creek, Claravale, Kakadu and Tolmer Falls colonies in one group, while the Pungalina colony formed a separate cluster (*F*_4,241_ = 68.01, *p* < 0.001) (Figure 9, Table S5). Validation using a random half of the dataset resulted in a low accuracy of correct assignment for chirp‐trills originating from Tolmer Falls (32%), Pine Creek (26%) and Kakadu colonies (16%), and moderate accuracy for Claravale (77%) and Pungalina colonies (75%), with an overall accuracy of correct assignment of 44.7%.

#### 3.3.2 Squabble

All acoustic measurement variables calculated for the squabble showed significant differences among colonies except for *F*_peak_ (Figure 8, Table S4). A DFA on squabble exemplars using all the acoustic variables except *F*_peak_ showed more variation among sites than a similar analysis of chirp‐trill vocalisations, but the squabble produced by the Pungalina colony still grouped separately from a cluster containing the remaining colonies (*F*_4,197_ = 122.4, *p* < 0.001). Within the latter group, the Pine Creek and Claravale bats produced squabble vocalisations that were only dissimilar from Kakadu vocalisations, and vocalisations from Tolmer Falls did not vary from the other northernmost colonies in this cluster (Figure 9, Table S5). Overall, the squabble was correctly assigned to the colony of origin 50.4% of the time (Pine Creek: 63%, Claravale: 52%, Pungalina: 57.1%, Kakadu: 78.9%, Tolmer Falls: 0%).

#### 3.3.3 Ultrasonic Social

All acoustic measurement variables differed significantly among sites for the ultrasonic social vocalisation (Figure 8, Table S4). Using DFA, ultrasonic social vocalisations from bats at Kakadu and Pungalina were relatively similar (Figure 9, Table S5), as were vocalisations from Claravale and Pine Creek colonies, while vocalisations from the Tolmer Falls colony formed a distinct cluster (*F*_4,208_ = 55.68, *p* < 0.001). Validation showed that 46.7% of vocalisations were correctly attributed to the colony of origin (Pine Creek: 65%, Claravale: 32%, Pungalina: 85%, Kakadu: 18%, Tolmer Falls: 25%).

#### 3.3.4 Echolocation

For echolocation calls, all variables showed significant differences among colonies (Figure 8, Table S4) except peak time (*F*_4,143_ = 0.4, *p* = 0.809). A DFA using all measurement variables except peak time showed that echolocation call structure only differed between Pine Creek and the other colonies (*F*_4,143_ = 25.13, *p* < 0.001) (Figure 9, Table S5). Levels of correct assignment were relatively high for the Pine Creek (73.9%) and Pungalina (81%) colonies, compared with Kakadu (42%), and there were no correct assignments for Claravale or Tolmer Falls. Overall, total correct assignments averaged 58.1%.

### 3.4 Correlation of Genetic Distance With Acoustic Distance

**FIGURE 10:**
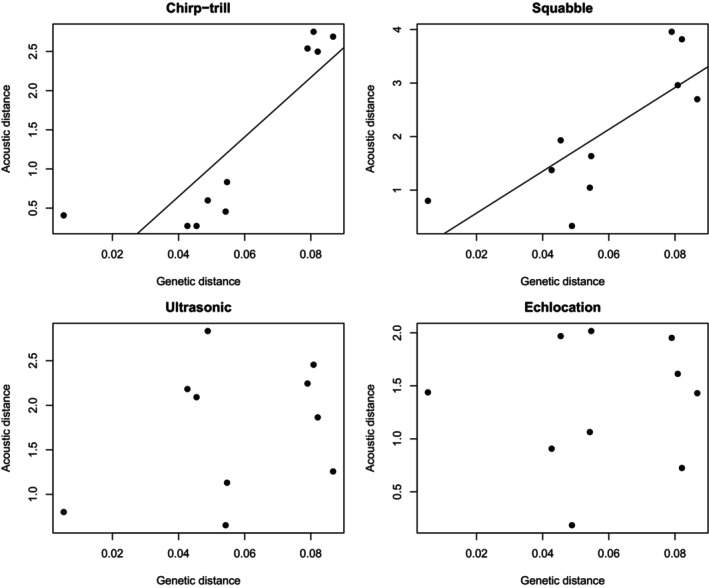
Scatterplots show that acoustic distance is correlated with genetic distance for the chirp‐trill and squabble but not the ultrasonic social vocalisation or echolocation call. The trend lines were produced using linear regression, but the correlation analysis was calculated using Mantel tests.

Genetic distance was positively correlated with acoustic distance for the chirp‐trill (*r* = 0.849, *p* = 0.060) and the squabble (*r* = 0.780, *p* = 0.061), and this correlation was marginally significant for both vocalisations; that is, colonies that were more genetically distinct also tended to produce chirp‐trills and squabbles that were more acoustically distinct (Figure 10). Acoustic distance and genetic distance were not correlated in the ultrasonic social vocalisation (*r* = 0.301, *p* = 0.182) or the echolocation call (*r* = 0.072, *p* = 0.381).

### 3.5 Correlation of Geographic Distance With Acoustic Distance

**FIGURE 11:**
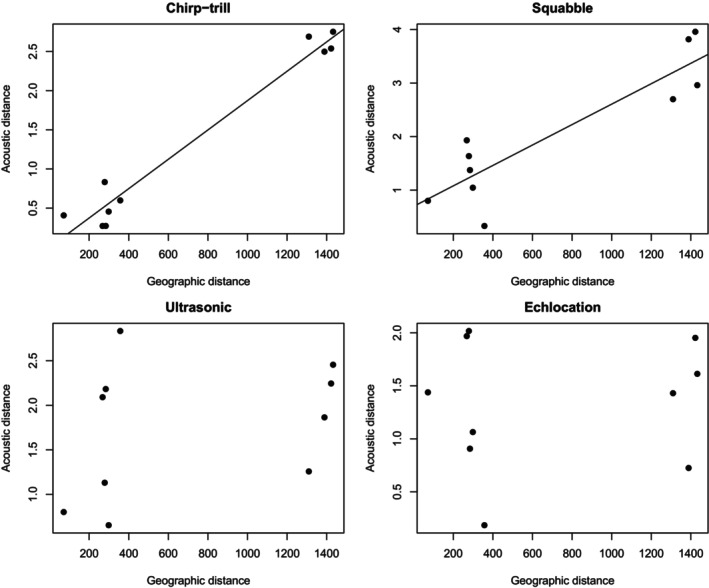
Scatterplots showing that acoustic distance is marginally correlated with geographic distance for the chirp‐trill and squabble, but no correlation was detected for the ultrasonic social vocalisation or echolocation call. Trend lines were produced using linear regression, but the correlation analysis was calculated using Mantel tests.

The acoustic distance of the chirp‐trill and squabble among geographically distinct colonies was marginally significantly correlated with geographic distance (*r* = 0.982, *p* = 0.077; *r* = 0.894, *p* = 0.074; respectively). The acoustic distance of the ultrasonic social vocalisation and the echolocation call was not significantly correlated with geographic distance (*r* = 0.318, *p* = 0.175; *r* = 0.107, *p* = 0.325; respectively) (Figure 11). Thus, the relationship between acoustic distance and geographic distance followed the same overall pattern as the relationship between acoustic distance and genetic distance, which is not surprising because genetic distance and geographical distance themselves were highly correlated (Figure 5).

### 3.6 Partial Mantel Tests

Because genetic and geographic distances were strongly correlated, partial Mantel tests were performed to assess whether the relationships between acoustic and genetic distances persisted after accounting for geography (Table S2). When geographic distance was controlled for, the correlations between acoustic and genetic distances were not significant for any call type (chirp–trill: *r* = −0.18, *p* = 0.48; squabble: *r* = −0.04, *p* = 0.50; ultrasonic social: *r* = 0.05, *p* = 0.38; echolocation: *r* = −0.05, *p* = 0.50). Similarly, when controlling for genetic distance, the correlations between acoustic and geographic distances were not significant (chirp–trill: *r* = 0.89, *p* = 0.17; squabble: *r* = 0.69, *p* = 0.14; ultrasonic social: *r* = 0.12, *p* = 0.43; echolocation: *r* = 0.09, *p* = 0.38).

### 3.7 Correlation of Morphological Distance With Acoustic Distance

Ear length was significantly correlated with squabble acoustic distance (*r* = 0.940, *p* = 0.021). Forearm length was marginally significantly correlated with ultrasonic social vocalisation acoustic distance (*r* = 0.391, *p* = 0.078). No morphometrics were correlated with the chirp‐trill or echolocation call (Data not shown).

## 4 Discussion

Our study assessing the extent of genetic, morphological, and acoustic divergence among five geographically dispersed colonies of the ghost bat shows that isolation by distance is occurring in this species, with colonies that are geographically further apart also being more genetically distant. In addition, ghost bats from Pungalina, the southernmost and most geographically distant of the five studied colonies, were larger in three morphometrics, while noseleaf length remained consistent among the colonies. We also identified acoustic divergence among colonies, albeit the processes affecting this divergence appeared to differ with vocalisation type. We identified weak correlations between geographic distance and acoustic distance, and between genetic distance and acoustic distance, for two social vocalisation types in the human‐audible range (‘chirp‐trill’ and ‘squabble’), suggesting that drift is acting on vocalisation structure to form dialects, which is consistent with the only other study that accounted for phylogenetic influences in bat social vocalisation divergence (Montero et al. 2018). In contrast, the lack of correlation detected between the divergence in the ultrasonic social vocalisation and echolocation calls with geographic or genetic distance suggests that these call types are subject to strong functional constraints that limit divergence among populations, consistent with the action of stabilising selection. Our study was limited to approximately 20% of the estimated global population (based on a total global population estimate of less than 7000 individuals; Threatened Species Scientific Committee 2016), and even at this fine scale, it clearly reveals dialect formation among colonies, suggesting that the comparison of social acoustic structure among the disjunct NT, Queensland and Western Australia populations of ghost bats would yield greater differences still.

Ghost bat colonies were more genetically distinct as geographic distance increased, indicating that isolation by distance operates within the NT metapopulation. At the small end of the scale, individuals captured at the Claravale and Pine Creek colonies, located 39 km apart, were genetically indistinguishable. This is consistent with the study from Worthington Wilmer et al. (1999) that found that the Pine Creek and Claravale colonies did not show significant differences in MtDNA or microsatellite alleles, prompting the authors to suggest (i) regular movement of individuals between colonies was occurring, or (ii) that Pine Creek was recently founded from the Claravale colony. More distant colonies, Tolmer Falls, Kakadu and Pungalina, all formed their own genetic clusters separate from the Claravale/Pine Creek cluster, indicating that gene flow is limited among those sites. This was also the finding of Worthington Wilmer et al. (1999) where sites' mitochondrial DNA differentiation was moderate but microsatellite differentiation was low; a genetic pattern consistent with male dispersal and high female philopatry. The greatest confirmed nightly travel distance by a ghost bat is approximately 48 km, based on a tracked female in the Katherine region, NT (Ruykys et al. in review), with similar distances recorded in the Pilbara region, Western Australia (Bullen et al. 2023). Limited studies on the seasonal movement of ghost bats recorded tagged male and non‐breeding female ghost bats up to 100 km from their roost of capture with some suspected of travelling up to 150 km over multiple nights (Douglas 1980; Toop 1985). This indicates that it is certainly possible for ghost bats to travel the 39 km between Claravale and Pine Creek. An inability to disperse is unlikely to be a factor in the lack of gene flow among the four northernmost colonies, suggesting that movement is limited by some other factor, such as a lack of intermediate day roosts between colonies to facilitate long‐distance travel. Social factors too may pose barriers to gene flow in this species; for example, while males may travel among colonies, they may have limited mating success with females other than those from the male's natal colony if mate choice is based on social call characteristics in this species.

Forearm and ear length were correlated with genetic and geographic distance. Forearm measurements are commonly used as a proxy for body size in bats (Fleming 1991; Storz et al. 2008). According to Bergmann's rule, individuals within a species that reside closer to the equator (i.e., in hotter climates) will be smaller than individuals further away, and this pattern is particularly prevalent in carnivores (McNab 1971). As a species that regularly consumes vertebrate prey including other mammals, the ghost bat appears to obey this rule with individuals from the cooler and drier locations in the south being larger than ghost bats from northern more tropical locations (Hand and York 1990). Although we did not account for latitude in our study, our most geographically distant colony, Pungalina, is also the most southern colony. Consistent with Bergmann's rule, ghost bats from Pungalina were significantly larger than the other four northern colonies in all metrics except nose leaf length. The nose leaf functions to direct echolocation calls (Schnitzler and Grinnell 1977), and so nose leaf shape is likely to be under a functional constraint.

Acoustic divergence was present among all colonies for each vocalisation type examined. For the chirp‐trill and squabble social vocalisations, acoustic distance was strongly correlated with both genetic and geographic distance, although these relationships were only marginally significant, suggesting that genetic or cultural drift has occurred because of geographic isolation. Because genetic and geographic distances were highly correlated (indicating isolation by distance), partial Mantel tests were performed to determine whether these associations between acoustic and genetic distances persisted after accounting for geography. When geographic distance was controlled for, the correlations were not significant, indicating that the apparent relationships between acoustic and genetic distances for the chirp–trill and squabble calls are largely explained by geographic separation among colonies rather than by direct genetic influences on call structure. The limited dispersal among colonies allows for stronger vertical (mother‐offspring) and horizontal (among colony members) transmission of information and reinforcement of particular characteristics of their communication system (Chen et al. 2009; Yoshino et al. 2008). Genetic drift has been implicated as a process of call evolution in birds, mice and frogs (Campbell et al. 2010; Graham et al. 2018; Irwin et al. 2008; Lee et al. 2016; Lipshutz et al. 2017; Sosa‐López et al. 2013). In bats, genetic drift has predominantly been investigated as a factor influencing geographic variation in echolocation calls (Armstrong and Coles 2007; Chen et al. 2009; reviewed in Jiang et al. 2015). In contrast, few studies have investigated the relationship between acoustic and genetic divergence in social vocalisations (Montero et al. 2018; Sun et al. 2020; Zhang et al. 2024) and tend to be biased towards species that develop these vocalisations through vocal production learning (Esser 1994; Boughman 1998; Esser and Schubert 1998; Davidson and Wilkinson 2002; Prat et al. 2015; reviewed in Knörnschild 2014).

The ultrasonic social vocalisation and echolocation call both showed significant differences among colony locations, but this divergence was not related to genetic, geographic or morphological distance, ruling out genetic drift as a significant process of dialect formation for these vocalisations. Instead, the lack of correlation with genetic or geographic distance suggests that these call types are highly conserved across colonies, consistent with strong functional constraints and stabilising selection acting on calls that serve essential navigational or prey‐detection roles. Both call types are produced nasally and share structural features closely tied to foraging and orientation performance, which likely limits their evolutionary flexibility. Echolocation call structure is tightly linked to the prey types and foraging habitat and thus is under stabilising selection, and bats can exhibit plasticity in the structure of their echolocation call depending on local differences in the environment (Berger‐Tal et al. 2008; Kalko and Schnitzler 1993). Pine Creek echolocation calls were significantly different from those of the other colonies, and we suggest that this is because vocalisations were recorded at Pine Creek deep within a mine shaft rather than at the cave opening as at other sites. As we specifically avoided acoustic measurements that may be confounded by echoes in our analysis (such as ‘entropy’), our findings suggest that ghost bats alter the structure of their echolocation call when inside the roost compared to at the roost entrance. Echolocation call structure should ideally be measured when bats are foraging, but this is very difficult in ghost bats because their echolocation calls have very low amplitudes, like those of the ‘whispering bats’ such as *Nyctophilus* species (Fenton 1982), and therefore not easily detected in foraging areas because of the short detection range.

We found that the ultrasonic social vocalisation could structurally be separated into three geographic groups: Southern Top End (Pine Creek and Claravale), Northern Top End (Tolmer Falls and Kakadu) and the Gulf (Pungalina). Ultrasonic social vocalisations are produced throughout the year but peak significantly during the parturition period (Hanrahan et al. 2019), and so we hypothesised that this vocalisation type may represent an ‘isolation call’ produced by ghost bat pups (Hanrahan et al. 2019). Many bat species produce ‘isolation calls’ – so called because they function to elicit maternal care and are often used to reunite mothers and pups in the roost (Knörnschild, Nagy, et al. 2012). Isolation calls change as the pup matures, developing into echolocation calls (de Fanis and Jones 1996; Sterbing 2002; Wang et al. 2014) or adult social vocalisations (Brown 1976; Esser and Schmidt 1989; Goymann et al. 1999; Knörnschild, Nagy, et al. 2012). In the ghost bat, the ultrasonic social vocalisation, like the echolocation call, is emitted nasally and is structurally similar to the echolocation call, although it varies more and is often lower in *F*_max_ (Hanrahan 2019). The geographic differences observed in the ultrasonic social vocalisation do not rule out the possibility that geographic isolation influences variation through genetic or cultural drift. However, our data suggest that, because this call shares production mechanisms with the echolocation call, it may be constrained by selection acting on the latter.

In sum, this study provides the first evidence of dialect formation in megadermatids and adds to the depauperate research of dialects in bats. Our study demonstrates clear acoustic differences in all vocalisation types examined among the study colonies, with these differences being greater among colonies than within colonies, indicating the formation of dialects among NT ghost bat colonies. While genetic (and geographic) and morphological distance appear to explain some of this difference, other mechanisms, such as cultural drift, may also be a factor and require further investigation. Our study highlights the importance of investigating multiple processes of call evolution when investigating dialect formation, including considering different hypotheses for functional‐distinct vocalisation types. We suggest that further studies are warranted to investigate how social factors may facilitate or inhibit gene flow and determine the extent of dialect formation across the entire range of the ghost bat.

## Author Contributions

**Nicola Hanrahan:** conceptualization (equal), formal analysis (equal), investigation (lead), methodology (equal), writing – original draft (lead). **Kyle N. Armstrong:** conceptualization (equal), formal analysis (supporting), methodology (supporting), supervision (equal), writing – review and editing (equal). **Christopher Turbill:** conceptualization (equal), formal analysis (supporting), methodology (supporting), supervision (equal), writing – review and editing (equal). **Anastasia H. Dalziell:** conceptualization (equal), formal analysis (supporting), methodology (supporting), supervision (equal), writing – review and editing (equal). **Justin A. Welbergen:** conceptualization (equal), formal analysis (supporting), methodology (supporting), resources (supporting), supervision (lead), writing – review and editing (equal).

## Ethics Statement

The study protocol used was assessed and approved by the Western Sydney University Animal Ethics Committee (ARA A11403) and conducted under a Northern Territory Permit to Interfere with Wildlife (number 51879). Biological samples were collected in Kakadu National Park under Commonwealth Areas Permit AU‐COM2017‐364.

## Conflicts of Interest

The authors declare no conflicts of interest.

## Supporting information


**Appendix S1:** ece373797‐sup‐0001‐AppendixS1.docx.

## Data Availability

The data that support the findings of this study are openly available in Dryad at https://doi.org/10.5061/dryad.kprr4xhjf.
