# Supplementary material for: Dialect Formation in Ghost Bats: Genetic, Geographic and Morphological Drivers of Social and Echolocation Call Divergence
Source: Ecol Evol. 2026 Jan 4;16(1):e72797. doi: 10.1002/ece3.72797 (PMC12765597; doi:10.1002/ece3.72797)
Supplement: Supplementary file 1 — Appendix S1: ece373797‐sup‐0001‐AppendixS1.docx. [file ECE3-16-e72797-s001.docx]

**Appendix S1: Lost in translation: dialect formation among colonies of the ghost bat in relation to geographic, morphological, and genetic distance**

**Figures**

**
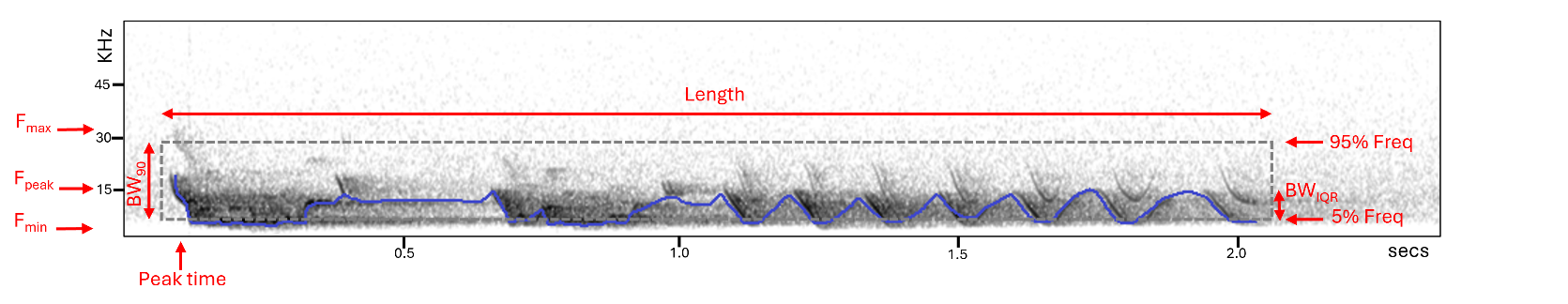
**

Figure S1 Spectrogram of the ghost bat’s chirp-trill vocalisation annotated with the measurement variables (red) used for analysis in Raven Pro. The blue line indicates the peak frequency contour. See Table 2 for measurement descriptions.

**
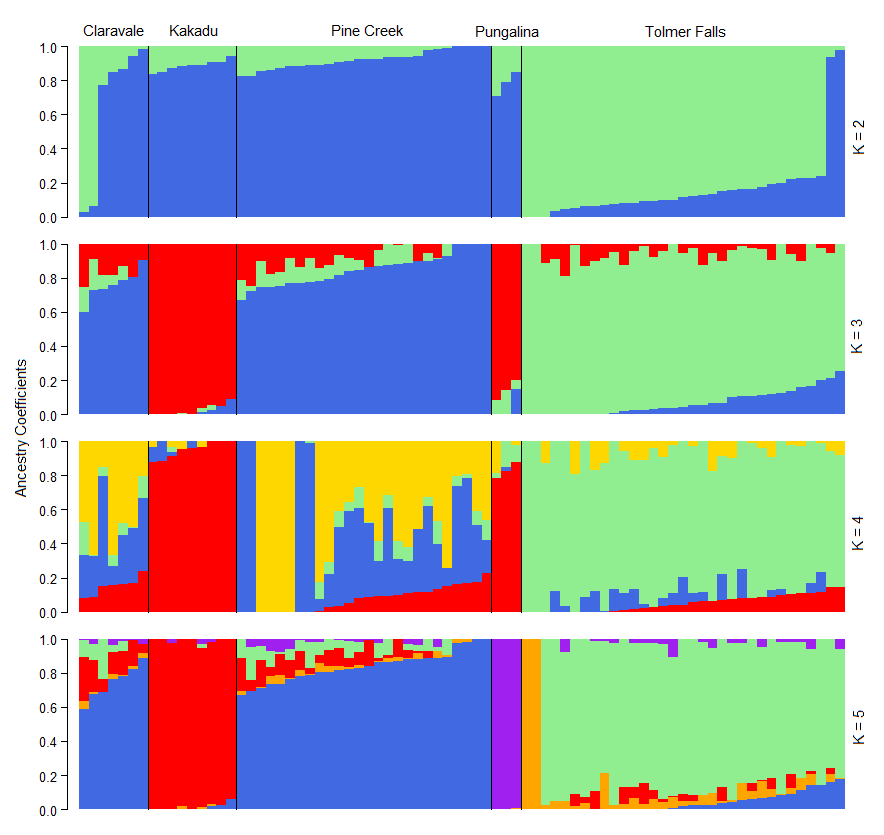
**

Figure S2 Graphical representation of ancestry coefficients for the dataset where K = 2, 3, 4 and 5 estimated using sNMF.

**Tables**

Table S1: Fixation index (*F*_ST_) values (grey below diagonal) showing genetic dissimilarities between colonies. Values can span from 0 to 1 with higher values indicating value greater genetic dissimilarity between colonies. Upper diagonal of table shows geographical distance (kilometres) between colonies.

| Study Site | Claravale | Pine Creek | Kakadu | Tolmer Falls | Pungalina |
| --- | --- | --- | --- | --- | --- |
| Claravale |  | 39 | 162 | 180 | 658 |
| Pine Creek | 0.002 |  | 148 | 139 | 681 |
| Kakadu | 0.023 | 0.025 |  | 225 | 654 |
| Tolmer Falls | 0.028 | 0.029 | 0.041 |  | 815 |
| Pungalina | 0.041 | 0.038 | 0.052 | 0.052 |  |

Table S2: Comparison of morphological measurements between male and female ghost bats. Welch’s two-sample t-tests were used to compare mean trait values between sexes. d = Cohen’s d (effect size). Only forearm length differed significantly between sexes (p = 0.003, d = 0.52).

| Trait | t | df | p | Female mean (mm) | Female SD | Male mean (mm) | Male SD | d |
| --- | --- | --- | --- | --- | --- | --- | --- | --- |
| Forearm | 3.01 | 111.26 | 0.003 | 104.3 | 3.3 | 102.6 | 3.3 | 0.52 |
| Noseleaf | 1.50 | 68.78 | 0.139 | 18.8 | 0.9 | 18.4 | 1.8 | 0.30 |
| Ear | 1.04 | 112.17 | 0.303 | 50.9 | 2.4 | 50.5 | 2.4 | 0.18 |
| Tragus | -0.91 | 99.68 | 0.364 | 22.4 | 1.7 | 22.7 | 2.0 | -0.16 |

Table S3: Mean morphological measurements for each colony. Asterisks (*) denote post hoc significance between pairs of colonies: *** = < 0.001, ** = < 0.01, * = <0.05. PC = Pine Creek, CL = Claravale, TF = Tolmer Falls, KA = Kakadu, P = Pungalina. Overall ANOVA significance of each morphometric variation among sites is provided.

| Colony |  | Forearm Length (mm) | Mean Ear Length (mm) | Mean Tragus Height (mm) | Mean Nose leaf Height (mm) |
| --- | --- | --- | --- | --- | --- |
| Pine Creek | Mean (mm) | 103.76 | 49.93 | 22.53 | 18.62 |
|  | Tukey Contrast | P*** | P*** | P*** | - |
| Claravale | Mean (mm) | 102.81 | 49.29 | 23.31 | 19.11 |
|  | Tukey Contrast | P*** | TF,KA*, P*** | P:*,TF** | - |
| Tolmer Falls | Mean (mm) | 103.56 | 51.03 | 21.87 | 18.52 |
|  | Tukey Contrast | P*** | P** | P***,CL** | - |
| Kakadu | Mean (mm) | 101.46 | 51.35 | 22.04 | 18.34 |
|  | Tukey Contrast | P*** | - | P*** | - |
| Pungalina | Mean (mm) | 108.88 | 53.56 | 24.92 | 19.21 |
|  | Tukey Contrast | All sites*** | CL,PC***, TOL** | CL*KA,PC,TF*** | - |
| ANOVA  Significance |  | F_4,136_ =12.23  p < 0.001 | F_4,136_ = 9.417  p < 0.001 | F_4,136_ = 10.76  p < 0.001 | F_4,136_ = 1.664  p = 0.162 |

Table S4: F-statistic and p values for acoustic measurements that showed significant differences among sites. NS – not significant.

| Acoustic measurement | Chirp-trill | Squabble | Ultrasonic social | Echolocation |
| --- | --- | --- | --- | --- |
| Bandwidth 90% | F_4,241_ = 28.24, p < 0.001 | F_4,197_ = 52.73, p < 0.001 | F_4,208_ = 16.48, p < 0.001 | F_4,143_ = 12.87, p < 0.001 |
| IQR bandwidth | F_4,241_ = 7.838, p < 0.001 | F_4,197_ = 56.41, p < 0.001 | F_4,208_ = 9.362, p < 0.001 | F_4,143_ = 7.167, p < 0.001 |
| Peak frequency | NS | NS | F_4,208_ = 14.34, p < 0.001 | F_4,143_ = 6.074, p < 0.001 |
| Length | NS | F_4,197_ = 8.911, p < 0.001 | F_4,208_ = 9.522, p < 0.001 | F_4,143_ = 2.802, p = 0.030^†^ |
| Min frequency | F_4,241_ = 4.025, p = 0.004 | F_4,197_ = 23.37, p < 0.001 | F_4,208_ = 9.777, p < 0.001 | F_4,143_ = 4.662, p = 0.001 |
| Max frequency | F_4,241_ = 52.45, p < 0.001 | F_4,197_ = 40.28, p < 0.001 | F_4,208_ = 21.95, p < 0.001 | F_4,143_ = 9.884, p < 0.001 |
| Peak time | NS | F_4,197_ = 2.633, p < 0.035 | F_4,208_ = 17.77, p < 0.001 | NS |

^†^Length of the echolocation call was significantly different among sites using one-way ANOVA, but p-values were not significant when adjusted for multiple post-hoc tests.

Table S5: Percent variance explained and variable loadings of the first two linear discriminants for each of the three social vocalisations and Echolocation call

|  | Chirp-trill LD1 | Chirp-trill LD2 | Squabble LD1 | Squabble LD2 | Ultrasonic social LD1 | Ultrasonic social LD2 | Echolocation call LD1 | Echolocation call LD2 |
| --- | --- | --- | --- | --- | --- | --- | --- | --- |
| Variance explained (%) | 90.0 | 5.7 | 89.3 | 8.8 | 68.6 | 16.3 | 61.2 | 25.9 |
| Variables | | | | | | | | |
| Bandwidth 90% | 0.450 | 0.505 | 0.446 | -0.952 | -0.622 | 0.120 | 0.942 | 0.229 |
| IQR bandwidth | 0.180 | -0.440 | 0.817 | 1.247 | -0.044 | -0.110 | -0.631 | -0.509 |
| Peak frequency | - | - | - | - | -0.291 | 0.642 | 0.020 | -0.022 |
| Length | - | - | -0.275 | 0.514 | -0.633 | 0.713 | 0.199 | 0.164 |
| Min frequency | 0.146 | 0.887 | 0.170 | 0.281 | -0.105 | 0.566 | 0.144 | -0.676 |
| Max frequency | 1.089 | -0.258 | 0.668 | -0.287 | -0.310 | -0.364 | 0.233 | -0.931 |
| Peak Time | - | - | 0.092 | -0.332 | 0.674 | 0.667 | - | - |
